# Supplementary material for: Fecal transplant allows transmission of the gut microbiota in honey bees
Source: mSphere. 2024 Aug 19;9(9):e00262-24. doi: 10.1128/msphere.00262-24 (PMC11423570; doi:10.1128/msphere.00262-24)
Supplement: Supporting information — Supplemental figures and tables. [file msphere.00262-24-s0001.pdf]

Fecal transplant allows transmission of the gut microbiota in honey bees.

Amélie Cabirol[1,*,#], Audam Chhun[1,*,#], Joanito Liberti[1,2], Lucie Kesner[1], Nicolas Neuschwander[1], Yolanda Schaerli[1], Philipp Engel[1]

[1] Department of Fundamental Microbiology, University of Lausanne, Switzerland

[2] Department of Ecology and Evolution, University of Lausanne, Switzerland

* Corresponding authors: amelie.cabirol@unil.ch, audam.chhun@unil.ch

[#] Authors contributed equally to this work

Supplementary Figures

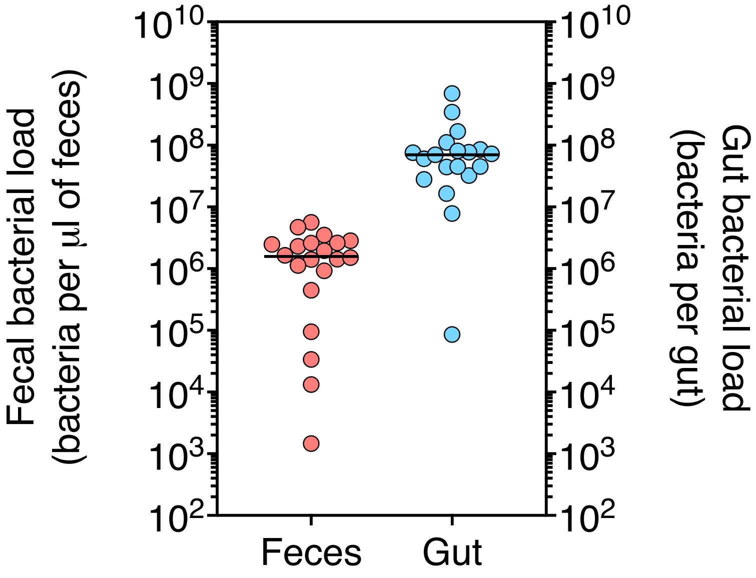

Supplementary Figure 1. Bacterial loads measured as copies of the 16S rRNA coding gene in the fecal and gut samples of nurse bees from generation no. 1. Horizontal bars represent the median values.

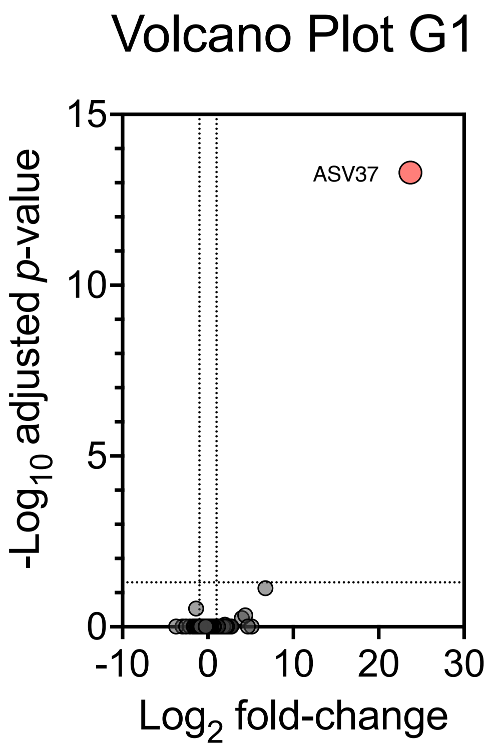

Supplementary Figure 2. Volcano plot presenting significance *versus* fold-change based on relative abundances of all amplicon sequence variants (ASVs) in the gut compared to the fecal samples of nurse bees from generation no. 1. The colored ASV was significantly different in DESeq2 analyses (FDR-corrected $P<0.05$). ASV37 corresponds to chloroplast DNA.

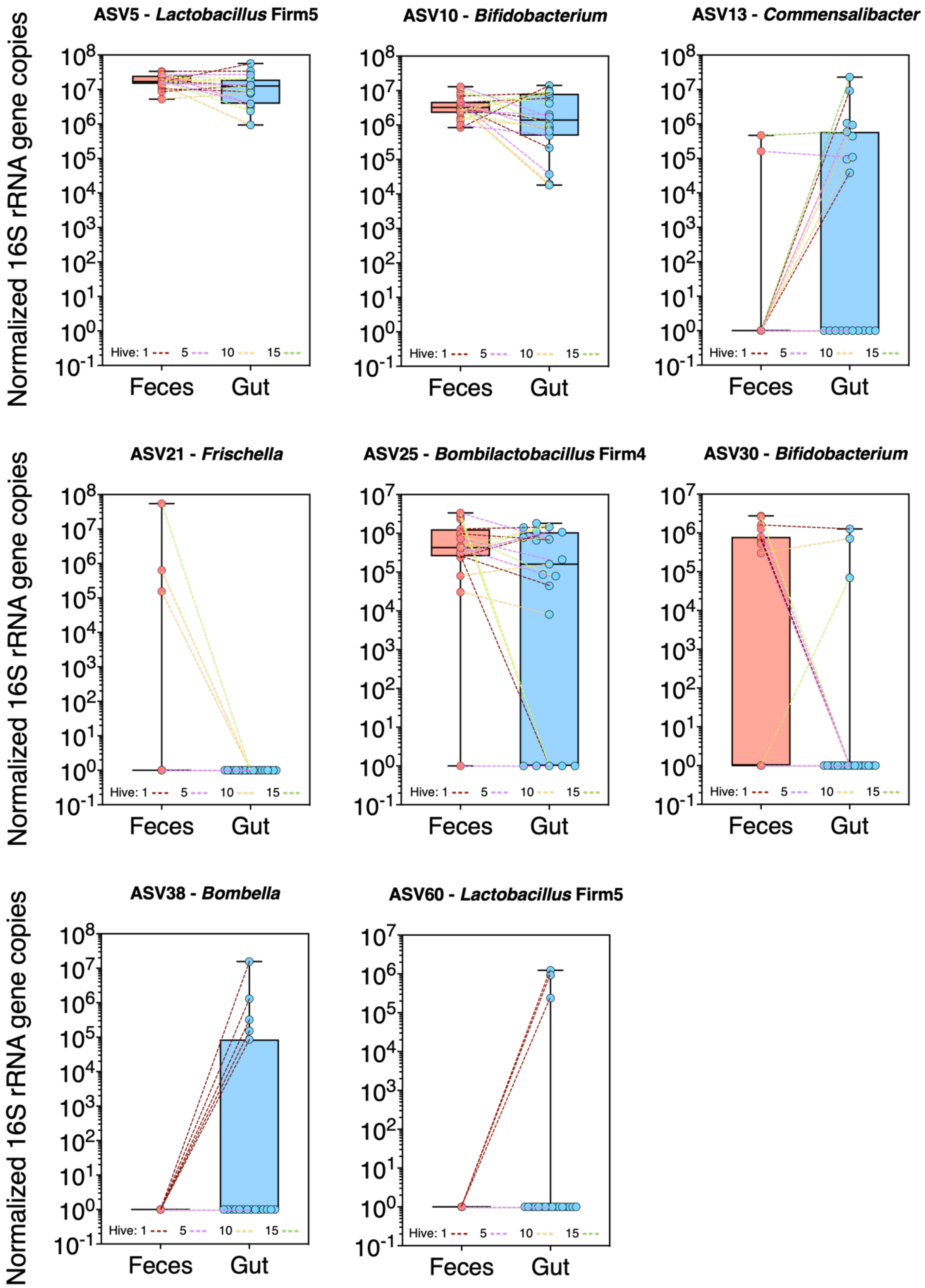

Supplementary Figure 3. Significantly different numbers 16S rRNA gene copies of various amplicon sequence variants (ASVs) in the gut of bees from generation no. 2, inoculated with either feces or gut homogenate.

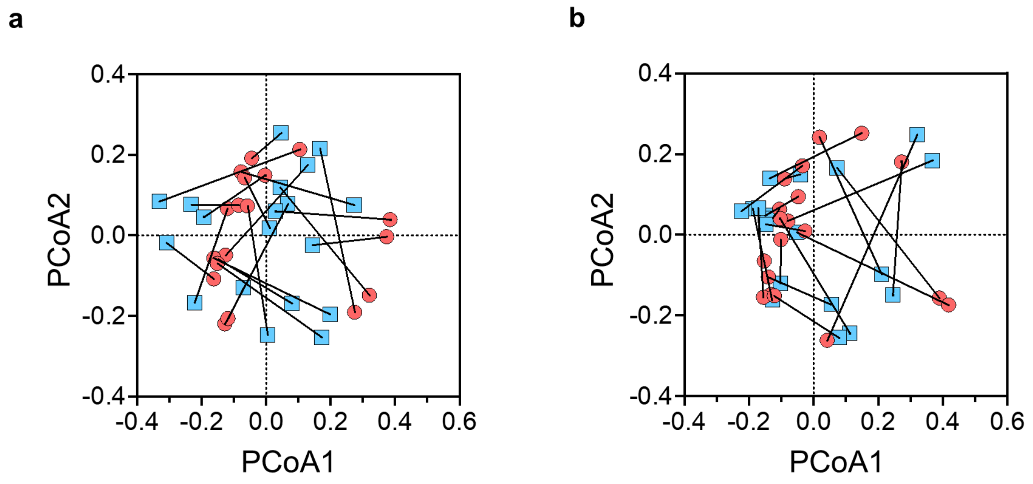

Supplementary Figure 4. Procrustes analysis of relative abundances of amplicon sequence variants (ASVs) in the fecal **(a)** or gut **(b)** samples of bees from generation no. 1 (red) against the gut of bees from generation no. 2 (blue) was obtained from PCoA and revealed no significant agreement of comparison.

Supplementary Table 1. ASVs detected in samples of generation no. 1 that are exclusively found either in gut or feces inoculum only. Listed ASVs are detected in less than two samples or at a relative abundance below 1%.

Generation no. 1			
**ASVs exclusive to gut inoculum**		**ASVs exclusive to feces inoculum**	
ASV100	Family_Mitochondria	ASV122	Brevibacterium
ASV111	Order_Chloroplast	ASV128	Methylobacterium-Methylorubrum
ASV121	Lactococcus	ASV140	Kytococcus
ASV126	Family_Mitochondria	ASV143	Family_Mitochondria
ASV127	Order_Chloroplast	ASV144	Pseudomonas
ASV131	Sphingomonas	ASV147	Bacillus
ASV135	Order_Rickettsiales	ASV148	Order_Chloroplast
ASV136	Psychroglaciecola	ASV160	Pseudocitrobacter
ASV137	Rickettsia	ASV175	Brevundimonas
ASV151	Family_Mitochondria	ASV176	Family_Mitochondria
ASV152	Geobacillus	ASV177	Acinetobacter
ASV153	Bacillus	ASV179	Friedmanniella
ASV161	Order_Chloroplast	ASV180	Thermincola
ASV162	Family_Erwiniaceae	ASV181	Peptostreptococcus
ASV163	Family_Pirellulaceae	ASV186	Leucobacter
ASV182	Snodgrassella	ASV187	Garicola
ASV183	Leptotrichia	ASV196	Dietzia
ASV188	Mycobacterium	ASV197	Kineococcus
ASV189	Family_Rhizobiaceae	ASV198	Roseomonas
ASV203	Rothia	ASV199	Novosphingobium
ASV204	Porphyrobacter	ASV201	Micrococcus
ASV205	Candidatus Aquiluna	ASV202	Patulibacter
ASV206	Family_Mitochondria	ASV87	Gilliamella
ASV207	Romboutsia	ASV90	Streptococcus
ASV208	Acinetobacter		
ASV28	Family_Mitochondria		
ASV61	Tatumella		
ASV66	Family_Enterobacteriaceae		
ASV69	Order_Chloroplast		
ASV74	Frischella		
ASV75	Tatumella		
ASV84	Gilliamella		
ASV85	Bombella		
ASV89	Family_Mitochondria		
ASV93	Acinetobacter		
ASV95	Staphylococcus		
ASV96	Order_Chloroplast		
ASV99	Family_Acetobacteraceae		

Supplementary Table 2. ASVs detected in samples of generation no. 2 that are exclusively found either in gut-inoculated or feces-inoculated bees only. Listed ASVs are detected in less than two samples or at a relative abundance below 1%

Generation no. 2			
ASVs exclusive to gut-inoculated bees		ASVs exclusive to feces-inoculated bees	
ASV113	Lactobacillus Firm5	ASV104	Order_Chloroplast
ASV150	Family_Mitochondria	ASV116	Family_Mitochondria
ASV31	Hafnia-Obesumbacterium	ASV149	Order_Chloroplast
ASV38	Bombella	ASV178	Class_Gammaproteobacteria
ASV41	Commensalibacter	ASV200	Class_Gammaproteobacteria
ASV43	Gilliamella	ASV21	Frischella
ASV45	Frischella	ASV33	Bombilactobacillus Firm4
ASV47	Order_Enterobacterales	ASV79	Bombilactobacillus Firm4
ASV50	Fructobacillus	ASV85	Bombella
ASV56	Bombilactobacillus Firm4	ASV92	Frischella
ASV60	Lactobacillus Firm5		
ASV63	Gilliamella		
ASV72	Lactobacillus Firm5		
ASV83	Bombilactobacillus Firm4		
ASV97	Bombilactobacillus Firm4		

Supplementary Video 1. Video (MP4 file format) showing feces sampling from one honey bee. The bee was CO_2-stunned and immobilized on ice for a few minutes before handling. Feces are collected into a sterile Eppendorf tube by gently pressing onto the bee abdomen, with a soft front-to-back motion.
